# A Boosting-Based Deep Distance Metric Learning Method

**DOI:** 10.1155/2022/2665843

**Published:** 2022-03-15

**Authors:** Zilong Li

**Affiliations:** ^1^School of Information Engineering, Xuzhou University of Technology, Xuzhou 221018, China; ^2^School of Computer Science & Technology, China University of Mining and Technology, Xuzhou 221116, China; ^3^Post-Doctoral Research Center, Onnes Power Technology Co., Ltd., Xuzhou 221003, China

## Abstract

By leveraging neural networks, deep distance metric learning has yielded impressive results in computer vision applications. However, the existing approaches mostly focus a single deep distance metric based on pairs or triplets of samples. It is difficult for them to handle heterogeneous data and avoid overfitting. This study proposes a boosting-based learning method of multiple deep distance metrics, which generates the final distance metric through iterative training of multiple weak distance metrics. Firstly, the distance of sample pairs was mapped by a convolution neural network (CNN) and evaluated by a piecewise linear function. Secondly, the evaluation function was added as a weak learner to the boosting algorithm to generate a strong learner. Each weak learner targets the difficult samples different from the samples of previous learners. Next, an alternating optimization method was employed to train the network and loss function. Finally, the effectiveness of our method was demonstrated in contrast to state of the arts on retrieving the images from the CUB-200-2011, Cars-196, and Stanford Online Products (SOP) datasets.

## 1. Introduction

In the past decades, distance metric learning has been applied effectively in image retrieval, face recognition, person re-identification, clustering, etc. It is now a hot topic in the field of computer vision. Thanks to the recent success of convolutional neural networks (CNNs), deep distance metric learning methods have attracted lots of attention [1].

Each deep distance metric aims to map training samples to features via CNNs. The mapping should narrow the distance between similar sample pairs and increase that between dissimilar sample pairs. To learn deep distance metrics, many approaches have been developed based on sample pairs [2, 3], triplets [2, 4], or quadruplets [5]. This study attempts to learn the simple similarity functions of sample pairs. The distance metric was defined as the Euclidean distance between sample pairs, which can be computed rapidly compared with other metrics.

Most of the existing methods of deep distance metric learning try to improve the single loss function based on a single distance metric. However, a single distance metric is insufficient to handle all the samples from the given data distribution. In fact, feature data are generally not distributed uniformly: the density varies from region to region in the data distribution [6, 7]. To solve the problem, some scholars resorted to ensemble technique and employed several learners to map each sample to multiple subspaces [8–10]. Nevertheless, these strategies do not support end-to-end training of the network and loss function of each weak learner. The lack of this training model suppresses the discrimination ability and increases the susceptibility of the metric to noise. The accuracy of deep distance metric learning could be further improved through joint training of the network and loss function.

**Figure 1:**
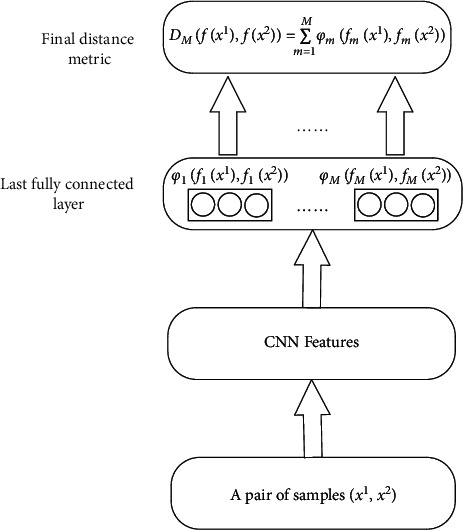
Structure of boosting-based deep distance metric.

This study aims to improve the adaptability of conventional deep distance metric between pairs of samples. The main idea is to divide the last fully connected layer of the CNN into multiple nonoverlapping groups (Figure 1), each of which is a separate feature mapping of the network. The distance metric of sample pairs mapped by one of the groups was evaluated by a piecewise linear function. Each group has a corresponding evaluation function, which is added as a weak learner to the boosting algorithm to generate a strong learner. This finally forms a multidistance metric ensemble. In addition, the same underlying feature representation, which was pretrained through experiments, was applied to the fully connected layers of all groups. In this way, the high computing cost of CNN training in the boosting framework was significantly reduced. In that framework, each learner reweights the training samples for successive learners, according to the gradient of the loss function. As a result, the successive learners would focus on difficult sample features, producing more suitable feature representations. The final ensemble output is a linear composition of multiple weak learners. Furthermore, the performance of the conventional distance metric was improved by introducing a piecewise linear function, which evaluates the similarity of sample pairs in distance metric learning. This facilitates the joint training of the network and loss function. Through the evaluation of various deep distance metric learning methods in the image retrieval task， it can be seen that Recall@1 of the proposed method is 4.2, 2.8, and 0.4 higher than that of the previous best score on CUB-200-2011， Cars-196, and SOP datasets, respectively. Experimental results show that the proposed method outperforms the comparison methods, while avoiding overfitting to a certain extent.

The contributions of our work are as follows:

1. The last fully connected layer of the CNN was used to form multiple groups of features, which was designed to form a distance metrics ensemble and formulated as a boosting problem. Then, an alternating optimization method was adopted to jointly train the network and loss function.
2. A piecewise linear function was employed as the evaluation function of the distance metric of sample pairs mapped by CNN and added as a weak learner to the boosting algorithm to generate a strong learner.

## 2. Literature Review

This section reviews the most closely related works out of the numerous publications on the hot topic of distance metric learning.

### 2.1. Deep Distance Metric Learning

Many methods employ a discriminative distance metric loss function to increase interclass distance and reduce intraclass distance [2, 4, 11–14]. For instance, contrastive loss is a popular tool of deep distance metric learning that minimizes the distance between the eigenvectors of positive sample pairs and widens the distance between the negative sample pairs [2, 3]. Based on contrastive loss, triplet loss creates a 3-tuple with a positive sample pair and a negative sample pair, in the light of the relative relationship between intraclass distance and interclass distance [2, 4], and ensures that the positive sample pairs are closer in the mapped feature space than the negative sample pairs. Many other loss functions have been extended from the above two losses, namely, histogram loss [15], quadruplet loss [5], N-pair loss [11], angular loss [12], and hierarchical triplet loss [16].

Taking a tuple of samples as training samples yields a huge amount of training data. Deep distance metric learning would be greatly enhanced by acquiring more effective samples. Recently, several scholars have designed sampling strategies to tackle hard and semihard negative mining [16–18]. For example, Xuan et al. [7] observed that easy positive samples help to preserve the intraclass difference and thus improve the generalization ability of triplet loss. However, the use of easy positive samples constantly underchallenges the metric, making the embedding space less discriminative.

### 2.2. Ensemble Learning

The methods above all strive to improve the loss function based on a single distance metric. However, it is difficult for them to adapt to all available data. Recently, ensemble learning, which iteratively trains an ensemble from several weak learners for the final prediction, has been incorporated to boost the generalization performance of deep metric learning.

Negrel et al. [8] explained how to use their boosting-based metric learning algorithm to compute hierarchical organizations of face databases. Kim et al. [9] introduced multiple attention-based learners for ensemble. Xuan et al. [10] grouped labels randomly to create a large family of related embedding models, which can serve as an ensemble. Sanakoyeu et al. [6] employed a divide-and-conquer strategy to divide the embedding space to several clusters and used each cluster to train a single learner.

### 2.3. Other Related Metric Learning

In addition, there are other types of distance metric approaches recently, such as sample selection, local metric, and hierarchical metric. Wu et al. [19] proposed a distance-weighted sampling procedure, which selects more informative and stable examples than traditional approaches, achieving excellent results in the process. Wang et al. [13] generalized tuple-based losses and reformulated them as different weighting strategies of positive and negative pairs within a minibatch. Roth et al. [20] proposed to learn the distribution for sampling negative examples instead of using a predefined one. Local metric learning methods [21, 22] learn a collection of Mahalanobis distance metrics, each operating on a different subset of the data obtained by K-means or Gaussian mixture clustering. From [23, 24], we learn a two-level category hierarchy by using coarse and fine classifiers. Ge et al. [16] proposed a hierarchical version of triplet loss that learns the sampling all together with the feature embedding.

Different from the above approaches, our approach realizes the end-to-end training of the network and loss function of each weak learner, thereby enhancing the accuracy of deep distance metric and reducing the probability of overfitting.

## 3. Methodology

### 3.1. Boosting-Based Deep Distance Metric Model

Let *X*=[*x*_1_, *x*_2_,…, *x*_*N*_] be *N* training sample pairs, each of which belongs to one of the two class labels *y*_*n*_ ∈ {−1, +1}. If the two samples belong to the same class, the pair is labeled *y*_*n*_ = +1 and called a positive sample pair; if the two samples belong to different classes, the pair is labeled *y*_*n*_ = -1 and called a negative sample pair.

We divide the last fully connected layer of the CNN into multiple nonoverlapping groups. The training sample pair *x*_*n*_=(*x*_*n*_^1^, *x*_*n*_^2^) is fed into the CNN to generate a eigenvector pair *f*_*m*_(*x*_*n*_)=(*f*_*m*_(*x*_*n*_^1^), *f*_*m*_(*x*_*n*_^2^)), which is extracted from the *m*th group of the last fully connected layer. So, the training sample pair *x*_*n*_ can be mapped to generate multiple groups of features, which was designed to form a distance metrics ensemble and formulated as a boosting problem.

Drawing on the idea of the boosting algorithm, multiple weak learners are adopted to produce a strong learner of distance metrics between the mapping values of training sample pairs. The weak learners are trained on reweighted samples, according to the gradient of the loss function. In general, we want to a set of weak learners and their corresponding boosting model:

(1) $$\begin{array}{c} D_{M}\left(f\left(x_{n}^{1}\right),f\left(x_{n}^{2}\right)\right)=\sum_{m=1}^{M}\varphi_{m}\left(f_{m}\left(x_{n}^{1}\right),f_{m}\left(x_{n}^{2}\right)\right), \end{array}$$

where *M* is the number of weak learners and *φ*_*m*_ is the distance metric evaluation function between the eigenvectors of the training sample pair mapped by the *m*th group of the fully connected layer.

**Figure 2:**
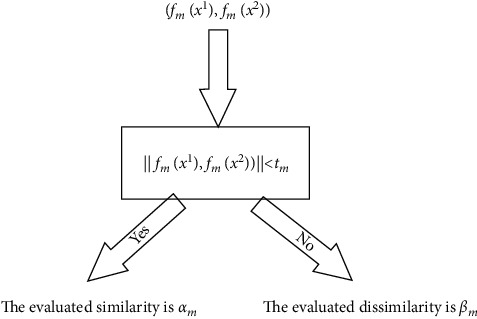
Operation of the evaluation function.

In the above formula, *φ*_*m*_ was used to quantify the similarity between two training samples, and it responds to this similarity based on whether the two samples should be considered to represent the same class. Therefore, a threshold was defined to deal with the distance metric between two training samples, and a piecewise linear function was adopted as the evaluation function. This function reduces the distance between similar training samples and increases that between dissimilar ones in the mapped space. The evaluation function can be defined as

(2) $$\begin{array}{c} \varphi_{m}\left(f_{m}\left(x_{n}^{1}\right),f_{m}\left(x_{n}^{2}\right)\right)=\left\{\begin{array}{c} \begin{array}{cc} \alpha_{m}, & \text{if }d\left(f_{m}\left(x_{n}^{1}\right),f_{m}\left(x_{n}^{2}\right)\right)<t_{m}, \end{array}\\ \begin{array}{cc} \beta_{m}, & \text{else}, \end{array} \end{array}\right. \end{array}$$

where d(*f*_*m*_(*x*_*n*_^1^), *f*_*m*_(*x*_*n*_^2^)) is a generic distance metric (the simple Euclidean distance), *α*_*m*_ and *β*_*m*_ are the evaluated similarity and dissimilarity between the two samples, respectively, and *t*_*m*_ is a distance metric threshold. If the Euclidean distance between two mapped training samples is smaller than the threshold *t*_*m*_, then the evaluation value is *α*_*m*_; otherwise, it is *β*_*m*_ (Figure 2).

In each round of boosting, a new weak learner is trained on the reweighted training set in the minibatch, according to the gradient of the loss function, and then added to form a strong learner. As demonstrated by Friedman [25], the training of a single learner can be written as a loss function minimization problem:

(3) $$\begin{array}{c} \arg\;\min\;\sum_{i=1}^{N}\ell \left(y_{i};D_{M-1}\left(x_{i}\right)+\varphi_{m}\left(x_{i}\right)\right), \end{array}$$

where *ℓ* is a loss function. Here, the exponential loss function *ℓ*(*y*_*i*_; *D*_*M*_(*x*_*i*_))=*e*^−*y*_*i*_*D*_*M*_(*x*_*i*_)^ is utilized. Inspired by Schapire and Singer [26], formula (3) can be rewritten as

(4) $$\begin{array}{c} \arg\;\min\;\sum_{i=1}^{N}w_{i}^{m}e^{-y_{i}\varphi_{m}\left(x_{i}\right)}, \end{array}$$

where *w*_*i*_^*m*^ is the weight of training sample *x*_*i*_ in iteration *m*. The weak learner is selected to minimize the loss function in each iteration to update the strong learner. Both *α*_*m*_, *β*_*m*_, and *t*_*m*_ of the distance evaluation function and *w*_*m*_^net^ of the *m*th group of the fully connected layer need to be optimized.

The proposed approach is easily integrated into some deep metric learning approaches, such as triplet loss, N-pair loss, and hierarchical triplet loss. However, for some loss functions, such as histogram loss and angular loss, it is not applicable and needs to be improved.

### 3.2. Joint Training

As to formula (3), we need attempt to jointly learn both the network and loss function. We note that its function was nonconvex, which was difficult to solve in general. Referring to Zhang et al. [27], this study applies an alternating optimization method to jointly train the network and loss function.

Since a learner needs to be optimized in each round of boosting, the optimization problem (4) was investigated, while fixing parameters *w*_*m*_^net^ of the *m*th group of the fully connected layer. Formula (4) can be decomposed into

(5) $$\begin{array}{c} \sum_{i=1}^{N}w_{i}^{m}e^{-y_{i}\varphi_{m}\left(x_{i}\right)}=\sum_{y_{i}=1\wedge \text{d}\left(x_{i}\right)<t_{m}}w_{i}^{m}e^{-\alpha_{m}}+\sum_{y_{i}=1\wedge \text{d}\left(x_{i}\right)\geq t_{m}}w_{i}^{m}e^{-\beta_{m}}+\sum_{y_{i}=-1\wedge \text{d}\left(x_{i}\right)<t_{m}}w_{i}^{m}e^{\alpha_{m}}+\sum_{y_{i}=-1\wedge d\left(x_{i}\right)\geq t_{m}}w_{i}^{m}e^{\beta_{m}}. \end{array}$$

Taking partial derivatives of formula (5) with respect to *α*_*m*_ and *β*_*m*_ and setting both to zero to optimize each parameter,

(6) $$\begin{array}{c} \alpha_{m}=\frac{1}{2}\log\left(\frac{\sum_{y_{i}=1\wedge d\left(x_{i}\right)<t_{m}}w_{i}^{m}}{\sum_{y_{i}=-1\wedge d\left(x_{i}\right)<t_{m}}w_{i}^{m}}\right), \end{array}$$

(7) $$\begin{array}{c} \beta_{m}=\frac{1}{2}\log\left(\frac{\sum_{y_{i}=1\wedge d\left(x_{i}\right)\geq t_{m}}w_{i}^{m}}{\sum_{y_{i}=-1\wedge d\left(x_{i}\right)\geq t_{m}}w_{i}^{m}}\right). \end{array}$$

After each iteration, the weights of the training sample pairs are updated using the exponential loss function:

(8) $$\begin{array}{c} w_{i}^{m+1}=w_{i}^{m}e^{-y_{i}\varphi_{m}\left(x_{i}\right)}. \end{array}$$

**Figure 3:**
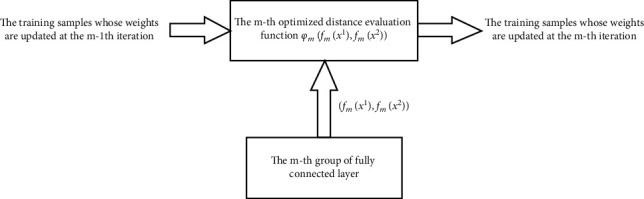
Optimization of the distance evaluation function with fixed parameters of the *m*th group of the fully connected layer.

Then, the weights of all training sample pairs are normalized. As shown in formulas (6) and (7), the parameters that affect the evaluation function are only related to *t*_*m*_, i.e., the optimal value obtained by the traversal method. If the training sample pairs are classified correctly, the weight of successive learners tends to be small; otherwise, the weight tends to be large. Hence, successive learners focus on different training sample pairs than previous learners, increasing the diversity among learners (Figure 3).

**Figure 4:**
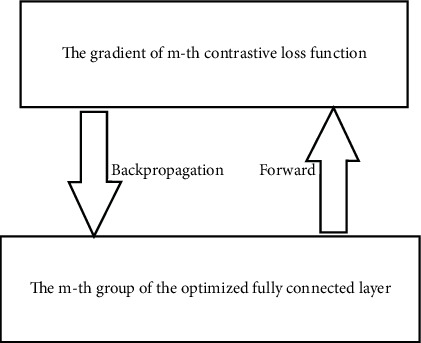
Optimization of the *m*th group of the fully connected layer with fixed parameters of the evaluation function.

The next step is to update parameters *w*_*m*_^*net*^ of the *m*th group of the fully connected layer, while fixing *α*_*m*_, *β*_*m*_, and *t*_*m*_ of the evaluation function. These parameters were trained with the contrastive loss function, using the standard backpropagation algorithm. In the forward process, the similarity distance metric is computed for each input training sample pair. In the backward process, the gradient of the loss function is iteratively propagated for each group (Figure 4).

For the contrastive loss function, the distance metric threshold *t*_*m*_ obtained through weak learning training serves as the distance margin of a negative training sample pair. Then, the contrastive loss function can be established as

(9) $$\begin{array}{c} \ell^{\prime }\left(x_{n}^{1},x_{n}^{2}\right)=\frac{1}{2}\left(y_{n}+1\right)\text{d}\left(f_{m}\left(x_{n}^{1}\right),f_{m}\left(x_{n}^{2}\right)\right)+\frac{1}{2}\left(1-y_{n}\right){\left[t_{m}-\text{d}\left(f_{m}\left(x_{n}^{1}\right),f_{m}\left(x_{n}^{2}\right)\right)\right]}_{+}. \end{array}$$

The training procedure is illustrated as Algorithm 1.

## 4. Experiments and Results' Analysis

To verify its effectiveness, our method for deep distance metric learning was tested on three standard datasets: CUB-200-2011, Cars-196, and Stanford online products (SOP). Following the standard protocol proposed by Oh Song et al. [2], each dataset was broken down into a training set and a test set. For the CUB-200-2011 dataset, 5,864 images in the first 100 classes were allocated to the training set and 5,924 images in the last 100 classes were allocated to the test set. For the Cars-196 dataset, 8,054 images in the first 98 classes were allocated to the training set and 8,131 images in the remaining 98 classes were allocated to the test set. For the SOP dataset, 59,551 images of 11,318 classes were allocated to the training set and 60,502 images in 11,316 classes were allocated to the test set.

The performance of our method in retrieving images from the above datasets was evaluated by Recall@K. For each retrieval task, the authors computed the percentage of the testing images whose top-K retrieved images contain at least one image with the same class label. The K value was set to K∈{1, 2, 4, 8, 16, 32} for CUB-200-2011 and Cars-196, and K∈{1, 10, 100, 1000} for SOP. Our method was implemented under the framework of TensorFlow. Following Oh Song's approach [2], GoogLeNet was adopted as the feature extractor. The batch size was fixed at 128 in all experiments.

**Figure 5:**
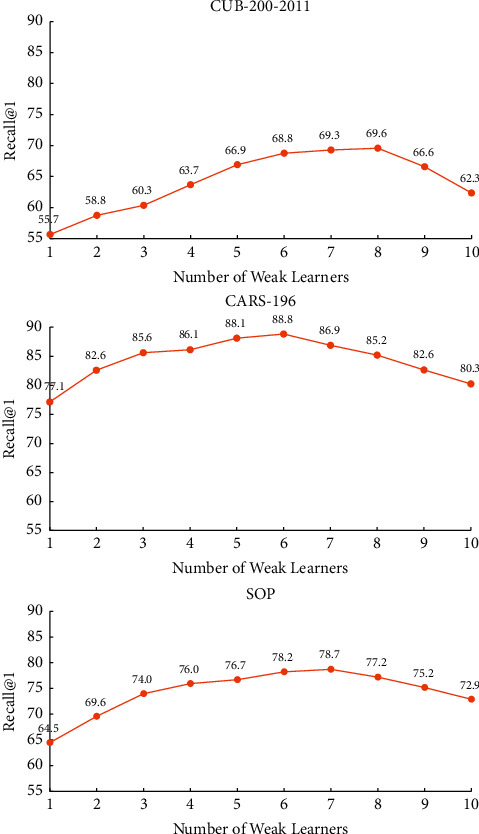
Influence of the number of weak learners over the performance of our method on different datasets.

Since the deep distance metric could be affected by the number of weak learners, the influence of that number on our method was observed through experiments on each of the three datasets. As shown in Figure 5, with the growing number of weak learners, the Recall@1 score first increased and then declined. The highest Recall@1 score was achieved at 8, 6, and 7 weak learners, for CUB-200-2011, Cars-196, and SOP, respectively. A possible reason is that the images in Cars-196 have relatively small variations, those in SOP face large view-point changes, and those in CUB-200-2011 feature a large pose variation and a strong background clutter. In the following experiments, the number of weak learners was set to 8, 6, and 7, for CUB-200-2011, Cars-196 and SOP, respectively.

**Figure 6:**
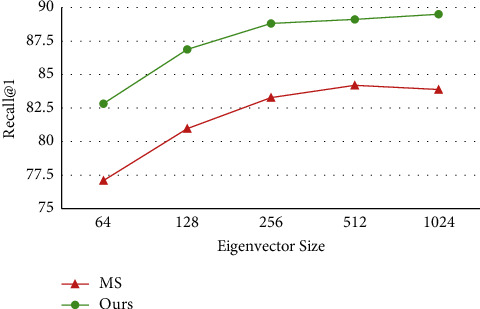
Retrieval performance (R@1) at different eigenvector sizes on Cars-196.

The eigenvector size also exerts a major effect on the deep distance metric. Hence, experiments were carried out on Cars-196 with 6 weak learners and different eigenvector sizes. Drawing on the work of Wang et al. [13], the eigenvector size was increased from 64 to 1,024.  Figure 6 compares the retrieval performance of our method with that of the multiscale (MS) method [13]. As shown in Figure 6, the retrieval performance of both methods gradually increased with the eigenvector size. Our method performed stably, when the size was equal to or greater than 256, and always outshined the MS. Hence, the eigenvector size was fixed at 256 in subsequent experiments.

**Figure 7:**
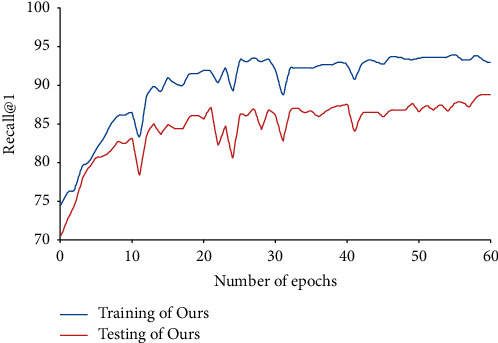
Training and testing results (R@1) on Cars-196.

Next, the training results and testing results were contrasted on Cars-196. As shown in Figure 7, the training R@1 only had a small gap from the testing R@1. On Cars-196, the R@1 score of the training set was around 93%, only 7% than that on the test set. This clearly shows that our method avoids overfitting.

**Figure 8:**
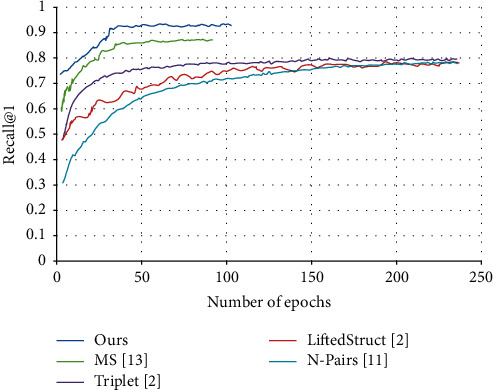
Convergence curves of different methods on Cars-196.

Figure 8 shows the convergence curves of our method and several state-of-the-art methods on Cars-196. In the first 40 epochs, our method reached the performance of the state of the arts and converged faster than the other methods. However, according to the trend of the curve in Figure 8, that is, number of epochs from 0 to 50, the convergence rate of ours model was not the maximum. However, on the whole, the convergence rate of our method was satisfactory. Except for the MS, the contrastive methods took hundreds of epochs to converge. Thus, the training time of our method was compared with that of the MS. On a single NVIDIA Tesla V100 GPU, the mean running time of our method was 24.36s per epoch on CUB-200-2011 and 40.29s per epoch on Cars-196, while that of the MS was 28.45s and 43.58, respectively.

Finally, the image retrieval efficiency of our method was compared with that of the state-of-the-art methods on CUB-200-2011 and Cars-196, respectively. The comparison results (Tables 1 and 2) show that our method outperformed these methods, including higher-order tuples such as LiftedStruct and N-Pairs, as well as angular loss and ensemble methods such as annotation-based expansion (ABE) and deep randomized ensembles for metric learning (DREML). In particular, on the challenging CUB-200-2011 dataset, our method led the best-performing state-of-the-art method by a large margin: 4.2% in R@1. On SOP, our method also attained the best performance (Table 3). On all the datasets, our method, with a low feature dimension, performed better than the existing methods, with high feature dimensions.

## 5. Conclusions

This study presents a deep distance metrics ensemble method based on boosting, which generates the final distance metric through iterative training of multiple weak distance metrics. Specifically, the last fully connected layer of the CNN was used to form multiple groups of features. The sample pairs were mapped by the CNN, and the distance between the mapped sample pairs was evaluated by a piecewise linear function. The function was added as a weak learner to the boosting algorithm to generate a strong learner. Then, an alternating optimization method was utilized to optimize the parameters of network and loss function. The effectiveness of our method was demonstrated on three datasets widely used in image retrieval tasks. The future research will further improve our method by cascading more models and combine our method with other loss functions.

## Figures and Tables

**Algorithm 1:**
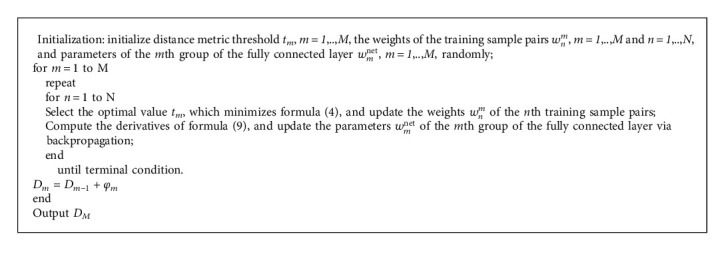
Training procedure of our method.

**Table 1:** Recall@K (%) on CUB-200-2011.

	CUB-200-2011					
R@	1	2	4	8	16	32
Contrastive [2]	26.4	37.7	49.8	62.3	76.4	85.3
Triplet [2]	36.1	48.6	59.3	70.0	80.2	88.4
LiftedStruct [2]	47.2	58.9	70.2	80.2	89.3	93.2
N-Pairs [11]	51.0	63.3	74.3	83.2	—	—
Angular loss [12]	54.7	66.3	76.0	83.9	—	—
HDC [28]	53.6	65.7	77.0	85.6	91.5	95.5
HTL [16]	57.1	68.8	78.7	86.5	92.5	95.5
ABE [9]	60.6	71.5	79.8	87.4	—	—
DREML [10]	63.9	75.0	83.1	89.7	—	—
MS [13]	65.7	77.0	86.3	91.2	95.0	97.3
SoftTriple [29]	65.4	76.4	84.5	90.4	—	—
Ours	69.6	80.3	87.2	93.5	97.6	98.8

*Note*. DHC and HTL are short for hybrid dilated convolution and hybrid transfer learning, respectively.

**Table 2:** Recall@K (%) on Cars-196.

	Cars-196					
R@	1	2	4	8	16	32
Contrastive [2]	21.7	32.3	46.1	58.9	72.2	83.4
Triplet [2]	39.1	50.4	63.3	74.5	84.1	89.8
LiftedStruct [2]	49.0	60.3	72.1	81.5	89.2	92.8
N-Pairs [11]	71.1	79.7	86.5	91.6	—	—
Angular loss [12]	71.4	81.4	87.5	92.1	—	—
HDC [28]	73.7	83.2	89.5	93.8	96.7	98.4
HTL [16]	81.4	88.0	92.7	95.7	97.4	99.0
ABE [9]	85.2	90.5	94.0	96.1	—	—
DREML [10]	86.0	91.7	95.0	97.2	—	—
MS [13]	84.1	90.4	94.0	96.5	98.0	98.9
SoftTriple [29]	84.5	90.7	94.5	96.9	—	—
Ours	88.8	94.2	96.9	98.4	99.1	99.6

**Table 3:** Recall@K (%) on SOP.

	SOP			
R@	1	10	100	1000
Contrastive [2]	42.0	58.2	73.8	89.1
Triplet [2]	42.1	63.5	82.5	94.8
LiftedStruct [2]	62.1	79.8	91.3	97.4
N-Pairs [11]	67.7	83.8	93.0	97.8
Angular loss [12]	70.9	85.0	93.5	98.0
HDC [28]	69.5	84.4	92.8	97.7
HTL [16]	74.8	88.3	94.8	98.4
ABE [9]	76.3	88.4	94.8	98.2
MS [13]	78.2	90.5	96.0	98.7
SoftTriple [29]	78.3	90.3	95.9	-
Ours	78.7	91.6	96.8	99.3

## Data Availability

The data used to support the findings of this study are available from the corresponding author upon request.
